# Screening for Compounds with Aromatase Inhibiting Activities from *Atractylodes macrocephala* Koidz

**DOI:** 10.3390/molecules16043146

**Published:** 2011-04-14

**Authors:** Hai Jiang, Jing Shi, Yuanyuan Li

**Affiliations:** 1 Department of Urology, First Affiliated Hospital, School of Medicine, Zhejiang University, No.79 Qingchun Rd., Hangzhou 310003, China; Email: jhai123@yahoo.com.cn; 2 Department of Pharmacy, Zhejiang Medical College, No.481 Binwen Rd., Hangzhou 310053, China

**Keywords:** aromatase, *Atractylodes macrocephala*, atractylenolide

## Abstract

Ten compounds were isolated from the dichloromethane extract of *Atractylodes macrocephala* and their aromatase inhibiting activities were tested using an *in vitro* fluorescent-based aromatase assay. The results indicated that atractylenolide I (**1**), atractylenolide II (**2**) and atractylenolide III (**3**) had inhibition ratios of 94.56 ± 0.70%, 90.93 ± 1.41% and 86.31 ± 8.46%, respectively, at a concentration of 10 μM. We conclude from our results that atractylenolide and its derivates may serve as potential aromatase inhibitors (AIs) and thus merit continued study in the future.

## 1. Introduction

**Figure 1:**
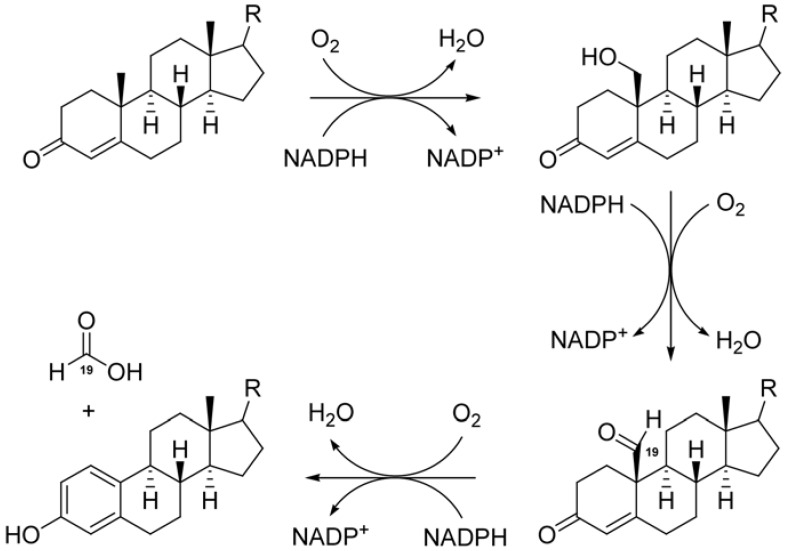
Estrone and estradiol biosynthesis mediated by aromatase (R = O, OH).

Breast cancer is one of the most commonly diagnosed types of cancer in women. In the United States, breast cancer is the second leading cause of death from cancer in women. About two-thirds premenopausal and three-quarters of postmenopausal breast cancer patients have estrogen-dependent cancer, which means estrogens play a critical role in stimulating breast cancer cell proliferation [1]. Therefore, antagonizing the action of estrogen can depress the development of breast cancer. According to the previous studies, there are two main approaches to control the pathological activity of estrogens [2]. The first is targeting the estrogen receptor directly, which results in the discovery of estrogen receptor antagonists tamoxifen and raloxifene [3,4]. The second is inhibiting aromatase (also called CYP19), a key enzyme that belongs to one of the subfamilies of cytochrome P450s and catalyzes the conversion of androgens to estrogens by altering the steroid enone A-ring to aromatic phenolic ring (Figure 1) [5]. From this point of view, aromatase is a seemly molecular target in the treatment of estrogen receptor sensitive breast cancer. Many aromatase inhibitors (AIs) were developed for the treatment of breast cancer, such as aminoglutethimide (AG), the first AI used in clinic and was ultimately withdrew due to its high toxicity and low selectivity [6]. Other effective AIs were formestane, exemestane, anastrozole and letrozole, which are remarkably potent and sufficiently selective drugs. AIs can be both steroidal and non-steroidal compounds [7].

The rhizomes of *Atractylodes macrocephala* Koidz (“Baizhu” in Chinese) is one of the most popular Traditional Chinese Medicines (TCMs), which has a long history of use for the treatment of splenic asthenia, anorexia, oedema, excessive perspiration and abnormal fetal movement. Previous chemical investigations on the rhizomes of *A. macrocephala* demonstrated that the main active constituents in them were sesquiterpenes and acetylenic compounds [8,9,10], which have been proven to possess anti-tumor and anti-inflammatory activities [11,12]. In the present study, ten compounds were isolated from the dichloromethane extract of *A. macrocephala*, which presented potent aromatase inhibiting activities.

## 2. Results and Discussion

**Figure 2:**
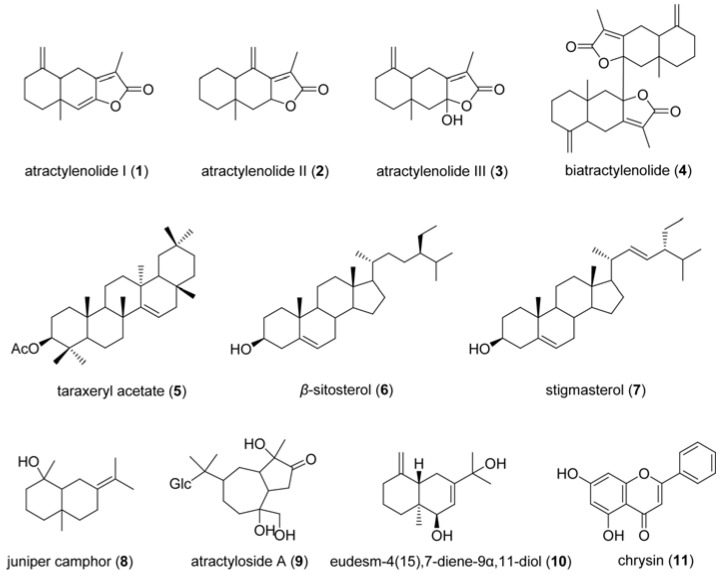
Chemical structures of compounds **1**-**11**.

The dichloromethane extract of *A. macrocephala* yielded ten known compounds (Figure 2): atractylenolide I (**1**), atractylenolide II (**2**), atractylenolide III (**3**), biatractylenolide (**4**), taraxeryl acetate (**5**), *β*-sitosterol (**6**), stigmasterol (**7**), juniper camphor (**8**), atractyloside A (**9**) and eudesm-4(15),7-diene-9*α*,11-diol (**10**). Their structures were elucidated on the basis of the results of NMR, MS and IR spectroscopic analysis and compared with the precious references [11,12,13,14]. 

**Figure 3:**
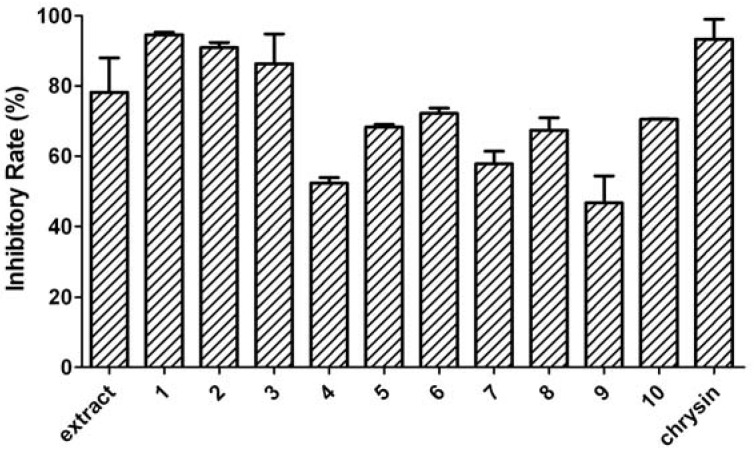
Aromatase inhibitory rates of the dichloromethane extract of *A. macrocephala*, compounds **1**-**10** and chrysin.

Since the dichloromethane extract of *A. macrocephala* showed aromatase inhibiting activity (the inhibitory rate was 78.17 ± 9.76%), we further tested the aromatase inhibiting activities of compounds **1**-**10** isolated from the extract. The results (Figure 3) indicated that all the compounds presented aromatase inhibiting activities. The aromatase inhibiting activities of compounds **1**-**3** were the most potent (the inhibitory rates were 94.56 ± 0.70%, 90.93 ± 1.41% and 86.31 ± 8.46%, respectively). Chrysin was tested as the positive control and its aromatase inhibitory rate was 93.30 ± 5.65%. Comparing the structures of ten isolated compounds, compounds **1**-**3** shared a common structural characteristic, which might play a critical role in their aromatase inhibiting activities. Therefore, it was suggested that atractylenolide and its derivates might serve as potential AIs and certainly merited continued and comparative study for the future.

## 3. Experimental

### 3.1. General

IR spectra were measured on a JASCO FTIR4100 spectrophotometer. ^1^H- and ^13^C-NMR spectra were recorded on a Bruker Ultrashield Plus 500 MHz spectrometer. The mass spectra were recorded using an LCQ Deca XP^plus^ ESI ion trap mass spectrometer (Thermo Finnigan, USA). Silica gel (300–400 mesh, Qingdao Chemical Group, China) was employed for column chromatography. The preparative HPLC experiments were performed on an Agilent 1200 HPLC system (Agilent Technologies, Germany) using Zorbax SB-C_18_ column (21.2 mm × 250 mm, 10 μm). Aromatase (Human CYP19 + P450 Reductase supersomes™) was purchased from BD Biosciences (Woburn, MA, USA). Chrysin was purchased from National Institute for the Control of Pharmaceutical and Biological Products (Beijing, China).

### 3.2. Plant materials

The rhizomes of *A. macrocephala* were purchased from the traditional Chinese medicine market at Hangzhou City, Zhejiang Province, P.R. China, in June 2008 and identified by the authors. A voucher specimen (No. AM080603) was deposited in the herbarium of Department of Pharmacy, Zhejiang Medical College.

### 3.3. Extraction and isolation

The dried rhizomes of *A. macrocephala* (10 kg) were extracted with 95% ethanol (1 L × 4) to give a crude ethanol extract. The residue was dissolved in H_2_O (1 L) and then extracted successively with petroleum ether (1 L × 3) and dichloromethane (1 L × 3). The dichloromethane part was subjected to column chromatography (silica gel), eluted with petroleum ether/ethyl acetate [100:5, 100:10, 100:20, 100:50, 100:100 (v/v)] and dichloromethane/methanol [100:10, 100:20 (v/v)] to afford seven fractions (Fr. A-G). Fr. A was further separated by preparative HPLC to yield compounds **1**, **2**, **4**, **5** and **8**. Fr. B was further separated by preparative HPLC to yield compounds **3 **and **6**. Fr. C was further separated by preparative HPLC to yield compound **7**. Fr. D was further separated by preparative HPLC to yield compound **10**. Fr. G was further separated by preparative HPLC to yield compound **9**.

### 3.4. Aromatase inhibition assay

Aromatase inhibition was evaluated by measuring the fluorescent intensity of fluorescein, the hydrolysis product of dibenzylfluorescein (DBF), by aromatase as described previously [15]. Compounds **1**-**10** and the dichloromethane extract of *A. macrocephala* were tested for their aromatase inhibiting activities. Briefly, the test sample (10 μL) was preincubated with a NADPH regenerating system (2.6 mM NADP^+^, 7.6 mM glucose-6-phosphate, 0.8 U/mL glucose-6-phosphate dehydrogenase, 13.9 mM MgCl_2_, and 1 mg/mL albumin in 90 μL of 50 mM potassium phosphate, pH 7.4) for 10 min at 37 °C. The final concentration of extract and each compound were 100 μg/mL and 10 μM, respectively. Then the enzyme and substrate mixture (0.8 μL DBF, 40 pmol/mL of aromatase, and 4 mg/mL albumin in 100 μL of 50 mM potassium phosphate, pH 7.4) were added to incubate for an additional 30 min at 37 °C. Finally, the fluorescence of reaction solvent was measured using infinite F200 scanning fluorescence plate reader (Tecan, Mannedorf, Switzerland) with excitation at 485 nm and emission at 535 nm. Chrysin was tested as positive control. Experiments were carried out three times on separate occasions.

## 4. Conclusions

In the present study, ten compounds were isolated from the dichloromethane extract of *A. macrocephala* and their aromatase inhibiting activities were tested using an *in vitro* fluorescence-based aromatase assay. Three compounds – atractylenolide I (**1**), atractylenolide II (**2**) and atractylenolide III (**3**) – showed potent aromatase inhibiting activities, with aromatase inhibition ratios of 94.56 ± 0.70%, 90.93 ± 1.41% and 86.31 ± 8.46%, respectively. In summary, atractylenolide and its derivates might serve as potential AIs and merit further study in the future.
